# How does the use of simultaneous contrast illusion on product-background color combination nudge consumer behavior? A behavioral and event-related potential study

**DOI:** 10.3389/fnins.2022.942901

**Published:** 2022-07-27

**Authors:** Minjing Peng, Yao Tong, Zhicheng Xu, Linli Jiang, Haiyang Huang

**Affiliations:** School of Economics and Management, Wuyi University, Jiangmen, China

**Keywords:** product-background saturation combination, purchase decisions, event-related potentials, N2, P3

## Abstract

Color, as one of the most critical visual factors influencing consumer decisions, has been widely used in e-commerce marketing. However, the effects of product-background saturation combination on consumers’ willingness to purchase products with different heaviness attributes (e.g., heaviness-positive products or heaviness-negative products) have not been conclusively determined. The current study demonstrated the effects of product-background saturation combination on product heaviness perception and its downstream consequences. Based on behavioral method, study 1 showed that a patch of color placed in a pale background (the saturation of the background is lower than the saturation of the color patch) was perceived as visually heavier than that in a colorful background (the saturation of the background is higher than the saturation of the color patch). Study 2 applied event-related potentials (ERPs) method to explore the underlying neural mechanisms of how the interactions between the presentation modes and the product types affect consumer decisions. Behaviorally, compared to the colorful background, the pale background would lead to a higher purchase rate for the heaviness-positive products, whereas the opposite results were found for the heaviness-negative products. Furthermore, for both the heaviness-positive and heaviness-negative products, a shorter reaction time would be observed in the pale background condition than in the colorful background condition. Neurophysiologically, the pale background would result in smaller N2 component and larger P3 component compared to the colorful background for the heaviness-positive products, while the reverse held for the heaviness-negative products. Smaller N2 component implies decreased perceptual conflicts and larger P3 component implies increased decision confidence, suggesting that e-retailers should present heaviness-positive products with pale backgrounds and heaviness-negative products with colorful backgrounds.

## Introduction

Due to advances in e-commerce, consumers have been exposed to large amounts of information when shopping online. Previous studies have indicated that consumers tend to rely on visually conveyed image information (e.g., product color, product background, and brand logo) to make quick decisions without textual descriptions of products (Bagchi and Cheema, 2013; Hagtvedt and Adam Brasel, 2017; Kunz et al., 2020; Huang et al., 2021). In marketing practice, color attracts consumers’ attention and provides them with information to evaluate the product. Typically, people develop an initial judgment about a person, environment, or product within 90 s. More importantly, 62–90% of their assessments rely on color alone (Singh, 2006). Notably, most of the existing studies on color in marketing have focused on hue and value, while saturation, one of the three dimensions of color, is still under-investigated (Labrecque et al., 2013; Labrecque, 2020).

**FIGURE 1:**
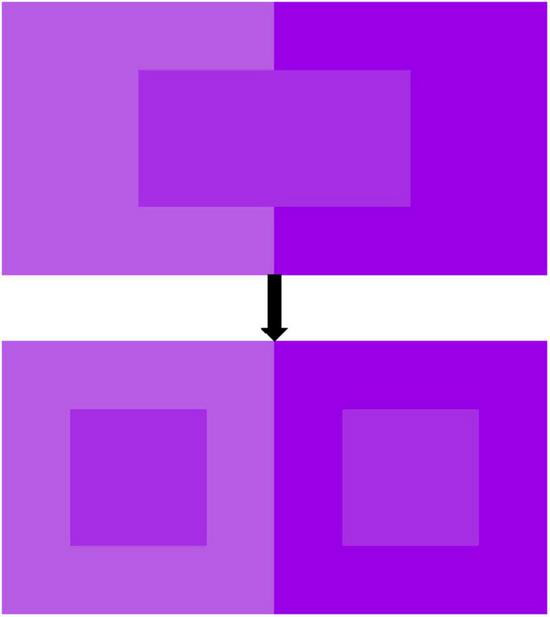
Simultaneous contrast.

The same color produces a different perception when it is adjacent to another different color. Dresp-Langley and Reeves (2012) demonstrated that true color on a general black background produces simultaneous brightness induction effects on two identical gray backgrounds and that simultaneous brightness also affects the perceived depth of color patterns. What’s more, they revealed that the color saturation of the background can determine the depth of the relative inducer, and also that the interaction of color saturation with contrast polarity can affect the background brightness, so that color saturation can modulate the figure-ground organization (Dresp-Langley and Reeves, 2014). The study by Hajdek et al. (2020) proposed the effect of simultaneous contrast on the perceptual properties of color and provided a design solution for real production. Figure 1 shows that placing a color adjacent to a highly saturated color decreases the saturation perception of that color; conversely, placing it adjacent to a low saturated color increases the saturation perception of that color. This phenomenon is known as simultaneous contrast (Chevreul, 1855; Labrecque, 2020). In addition, previous studies have shown that saturation is positively correlated with perceived heaviness (Alexander and Shansky, 1976; Walker et al., 2010). Therefore, we postulate that when one of two identical patches of color is surrounded by a desaturated background (the saturation of the background is lower than the saturation of the color patch), it appears heavier; however, when the other one is surrounded by a saturated background (the saturation of the background is higher than the saturation of the color patch), it appears lighter.

The impact of color on consumer behavior has been previously evaluated (Gorn et al., 2004; Bagchi and Cheema, 2013; Hagtvedt and Adam Brasel, 2017; Hagtvedt, 2020). For example, Bagchi and Cheema (2013) reported the effect of red backgrounds on consumers’ willingness to pay under different selling mechanisms. In auctions, the red background increased consumer markup behaviors; in contrast, it resulted in less bids by consumers during negotiations. Besides, higher product saturation allows consumers to have a larger perceived product size. Furthermore, consumers’ willingness to pay for products with high saturation is higher when their usage is targeted at larger sizes (Hagtvedt and Adam Brasel, 2017). It should be noted that prior studies have only focused on effects of a single carrier’s (product or background) color on consumer decisions. In real life, however, product and background colors are often presented together. Recently, studies have been aimed at elucidating the influence of color combination of the product and its background on consumer behaviors (Reynolds-McIlnay et al., 2017; Huang et al., 2020; Sharma, 2021). Reynolds-McIlnay et al. (2017) suggested that when products are neatly displayed, consumers exhibit a greater preference for the product with a high product–environment brightness contrast. Huang et al. (2020) showed that the use of similar colors for products and backgrounds improves product evaluations for functional products, while the use of contrasting colors for products and backgrounds improves product evaluations for sensory-social products. However, the effects of product-background saturation simultaneous contrast on consumer behaviors have not been conclusively determined. To fill this gap, we introduced research on heaviness.

Heaviness, as a product attribute, is not commonly preferred. For some products, heaviness is a positive attribute (when it is related to durability or richness), while for others, it is a negative attribute (when it is related to portability) (Deng and Kahn, 2009). For products with different heaviness attributes, consumers’ perceptions regarding product heaviness influences consumer behaviors. For instance, Deng and Kahn (2009) demonstrated that placing product image at the “heavier” locations (i.e., bottom, right, and bottom right) increases consumers’ perceptions of the product heaviness, thus enhancing the evaluation of heaviness-positive products (products for which heaviness is considered as a positive attribute). However, they observed a reversal of downstream effects for heaviness-negative products (products for which heaviness is considered as a negative attribute). More recently, Sharma and Romero (2020) indicated that product shadows also increase consumers’ perceived product heaviness and enhance consumer preferences for products for which heaviness is viewed as a positive attribute. Additionally, in different contexts, consumers have different expectations for product heaviness attributes. For example, if consumers want a durable laptop, they will choose the one with a dark color that looks heavy. In contrast, if they want a portable laptop, they will choose the one with a light color that looks light (Hagtvedt, 2020). Given the above-mentioned assumption that the perceived heaviness of a product will be increased when it is surrounded by the desaturated (vs. saturated) background, we hypothesize that the desaturated background increases consumers’ willingness to buy than the saturated background for the heaviness-positive products, whereas the reverse would be held for the heaviness-negative products. Nevertheless, there is neither behavioral nor psychological evidence to test this hypothesis in the existing literature.

**FIGURE 2:**
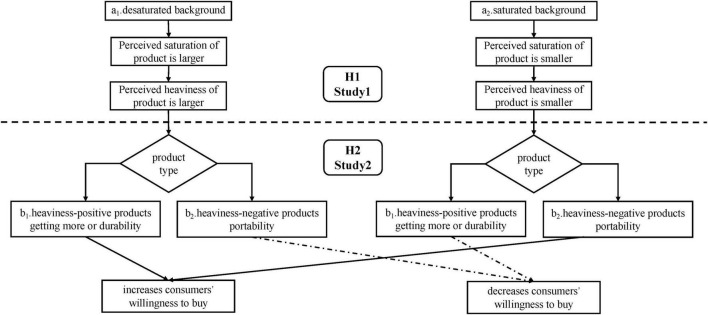
Diagram of theoretical model.

The current study explored how the use of simultaneous contrast illusions on product-background color combinations contributes to consumer behaviors. As shown in Figure 2, according to the principle of simultaneous contrast, the level of background saturation of a product affects the perceived saturation of the product and then affects the perceived heaviness of the product. The increased product heaviness perceptions enhance consumers’ willingness to purchase heaviness-positive products, and conversely, the decreased heaviness perceptions increase consumers’ willingness to purchase heaviness-negative products. We tested our hypotheses separately by conducting Study 1 and Study 2. In Study 1, we assessed the effects of simultaneous saturation contrast on perceived heaviness by behavioral method. In Study 2, we applied event-related potentials (ERPs) method to investigate the impacts of product-background saturation combination and product type on consumer decisions. To achieve our goal, we selected heaviness-positive and heaviness-negative products, and each product had two presentation modes (i.e., saturated background and desaturated background). In summary, this study involved four conditions, heaviness-positive product-desaturated background, heaviness-positive product-saturated background, heaviness-negative product-desaturated background and heaviness-negative product-saturated background. Based on previous literature on purchase decisions, this study focused especially on the N2 and P3 components.

N2, a negative component with a prefrontal-central region distribution, peaks at approximately 200–400 ms after stimulus onset (Folstein and Van Petten, 2007; Dickter and Bartholow, 2010). N2 reflects conflict and mismatch (Folstein and Van Petten, 2007). A larger N2 component is elicited when physical properties of two stimuli do not match (Tian et al., 2001; Mao and Wang, 2008; Han et al., 2015). Based on the S1-S2 paradigm, Han et al. (2015) showed that a more negative N2 component is induced when S1 and S2 differ in color, shape, and direction. Furthermore, the N2 component is evoked by perception conflicts (Ma et al., 2007, 2010, 2021; Shang et al., 2017; Song et al., 2020, 2022). Wang et al. (2016), for instance, asked participants to make purchase decisions based on product ratings and sales and found that the low product rating condition resulted in higher response conflicts, as reflected by the larger N2 amplitude. Jin et al. (2017) showed that negative framing messages induce greater cognitive conflicts and evoke greater N2 components than positive framing messages. In the current study, a product surrounded by the desaturated background is perceived to be heavier than the same product surrounded by the saturated background and will enhance consumer preferences for the heaviness-positive product. However, the reverse holds for the heaviness-negative product. That is, the desaturated background is matched with the heaviness-positive product, while the saturated background is matched with the heaviness-negative product. Therefore, we assume that the saturated background evokes a more negative N2 component compared to the desaturated background for the heaviness-positive product. Conversely, the opposite outcome is observed for the heaviness-negative product.

P3, a positive component maximal over central-parietal regions, typically arises at around 300–500 ms after stimulus onset. It indicates the event classification activities in the working memory (Azizian et al., 2006; Ma et al., 2008). A target-detection experiment by Azizian et al. (2006) revealed that compared to other stimuli, stimuli that were similar in perception to the targets elicit a more positive P3 component. Similarly, previous studies on brand extension indicated that a greater fit between the extension product and the parent brand results in a larger P3 component (Ma et al., 2008; Shang et al., 2017). Moreover, P3 also reflects decision difficulty and confidence. Past research suggested that a smaller P3 component is induced by greater difficulties or lack of confidence (Nieuwenhuis et al., 2005; Qin et al., 2009). For instance, Xie et al. (2016) found that consumers feel more difficult and have no confidence in the relatively consistent review contexts (vs. the absolutely consistent review contexts), as evidenced by a less positive P3 amplitude. In the current study, compared to the saturated background, the desaturated background is more desirable to consumer preference for the heaviness-positive product, which may lead to a better decision experience, whereas the reverse holds for the heaviness-negative product. Thus, we infer that the desaturated background results in a higher perceived fit and increased decision confidence compared to the saturated background for the heaviness-positive product, as reflected by a more positive P3 component. In contrast, the opposite result will be observed for the heaviness-negative product.

As discussed above, this study aims to mine how product-background saturation simultaneous contrast impacts consumer decisions by using behavioral and ERP measures. Based on previous studies, we propose the following hypotheses: (1) A patch of color surrounded by the desaturated background is perceived to be heavier than the same patch of color surrounded by the saturated background; (2) An attenuated N2 and an increased P3 are triggered by the desaturated background than by the saturated background for the heaviness-positive product, whereas the reverse holds for the heaviness-negative product.

## Study 1: A behavioral experiment

### Method

**FIGURE 3:**
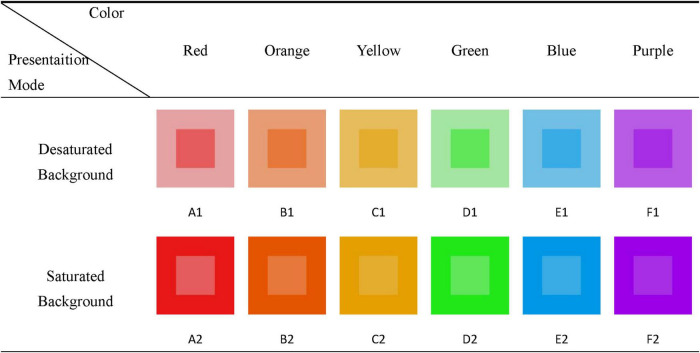
Two versions of the presentation mode (desaturated background and saturated background).

In Study 1, 119 participants from Wuyi University participated in a single-factor experiment in which presentation mode (desaturated background vs. saturated background) was manipulated as a within-subject factor in exchange for course credit. All participants had normal visions and were not color blind. As shown in Figure 3, two versions of the presentation mode were developed by manipulating background saturation of color patches. Furthermore, in accordance with the study by Hagtvedt and Adam Brasel (2017), we deliberately selected six colors, including red, orange, yellow, green, blue, and purple. The Photoshop software was used to process all stimuli and ensured consistency of brightness for each stimulus pair with the same hue. All the stimuli and their color specification related to Study 1 are presented in Supplementary Table 1.

This experiment was conducted on sojump.com, a specialized data collection website in China. Each participant randomly evaluated the heaviness of color patches in desaturated and saturated background, one at a time. The order of the two different presentation modes was counterbalanced. After viewing each stimulus, they were asked to evaluate visual perceived heaviness of the middle patches of color on two seven-point Likert item measures [1 = “extremely light/feathery,” and 7 = “extremely heavy/hefty” adapted from Deng and Kahn (2009)]. Finally, color blindness test and demographic questions were answered by the participants.

### Results

**FIGURE 4:**
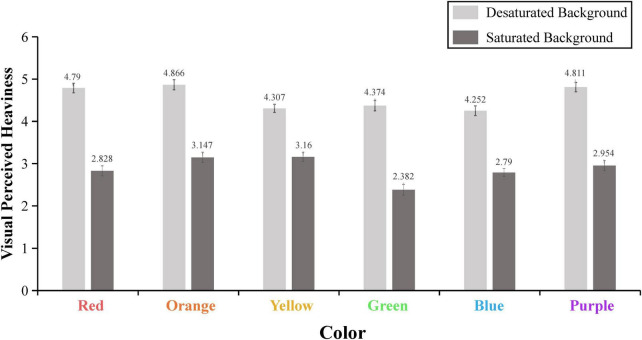
The presentation mode of a patch of color determines its visual heaviness. The error bars reflect the standard error of the mean.

We used the average of two scales to form a heaviness perception score for each middle patch of color. A repeated measure ANOVA was performed with presentation mode as a within-subject factor on this score. SPSS 25.0 was used for statistical analyses. The results revealed a significant main effect of presentation mode [*M*_desaturated_ = 4.567, *M*_saturated_ = 2.877; *F*(1,118) = 166.130, *p* < 0.001, $\eta_{P}^{2}$ = 0.585]: the patch of color surrounded by the desaturated background was perceived as visually heavier than that surrounded by the saturated background. In addition, this effect was observed for all the colors we have chosen, as shown in Figure 4 and Table 1.

**TABLE 1:** Statistical analysis results with two conditions for six colors.

Color	Desaturated background		Saturated background		*F*	*p*	$\eta_{P}^{2}$
	*M*	SE	*M*	SE			
Red	4.790	0.113	2.828	0.119	115.625	<0.001	0.495
Orange	4.866	0.119	3.147	0.122	93.542	<0.001	0.442
Yellow	4.307	0.097	3.160	0.109	50.456	<0.001	0.300
Green	4.374	0.124	2.382	0.127	102.292	<0.001	0.464
Blue	4.252	0.115	2.790	0.096	99.383	<0.001	0.457
Purple	4.811	0.111	2.954	0.119	132.661	<0.001	0.529

### Discussion

In Study 1, using a patch of color, we established that the saturation of a color patch’s background relative to itself determined its perceived heaviness. To enhance the external validity of this study, we used six colors (red, orange, yellow, green, blue, and purple). Subsequently, we designed two backgrounds (desaturated background and saturated background) for each small color patch. The results indicated that the middle color patch in the desaturated background was considered to be heavier than the middle color patch in the saturated background. More importantly, our results demonstrated that the effects of simultaneous contrast in color saturation on the perceived heaviness persisted regardless of hue.

Previous studies have established that size perception has an influence on consumer decisions. For example, Petit et al. (2018) found that placing food in a smaller container results in a relatively larger share illusion and improves consumer purchase intentions. This research applied the Delboeuf illusion (Delboeuf, 1865) to the marketing field. Following this logic, we believe that consumers’ perceptions of heaviness will also turn into their product preferences. Specifically, consumers’ purchase intentions will be increased when the perceived heaviness matches their expectations. Therefore, in Study 2, we applied the simultaneous saturation contrast to the product and directly tested the effects on consumer decision by using ERPs.

## Study 2: An event-related potential experiment

### Participants

Thirty-eight right-handed students with normal or corrected-to-normal visions from Wuyi University were recruited in this study. None of them was color blind or had any history of psychiatric or neurological disorders. Before the formal experiment, all participants were asked to provide written informed consents. All participants were paid ¥50 in return for their participation. Due to excessive artifacts during EEG data recording, one participant was excluded from the analysis. Therefore, data from 37 valid participants were analyzed (18 males, mean age = 21.6 years, SD = 2.1).

### Experimental stimuli

We designed a 2 (product type: heaviness-positive product vs. heaviness-negative product) × 2 (presentation mode: desaturated background vs. saturated background) experiment. For the product types, we selected six heaviness-positive products (laundry detergent, shampoo, beverage, potato chips, suitcase, and washing machine) and six heaviness-negative products (kettle, lamps, laptop, speaker, camera, and rucksack) as the stimulus materials based on the following two reasons: (1) The above products are common in people’s lives, therefore, participants are familiar with them; (2) Previous studies on the products’ heaviness properties frequently selected the above products as the objects of study (Deng and Kahn, 2009; Hagtvedt, 2020; Sharma and Romero, 2020; Choe et al., 2021). In addition, previous studies have suggested that the valence assigned to product attributes may be driven by consumers’ current mood or whether the attribute is consistent with a salient goal (Markman and Brendl, 2000; Adaval, 2001). Therefore, based on previous studies (Deng and Kahn, 2009; Sharma and Romero, 2020), we manipulated situational cues (heaviness-positive products: getting more or durability; heaviness-negative products: portability) to highlight product heaviness attribute. Two versions of the presentation mode (desaturated background vs. saturated background) were designed by manipulating the product background saturation. Specifically, for the same product, we designed different background saturation levels. One background saturation level is higher than the product saturation (saturated background) and the other background saturation level is lower than the product saturation (desaturated background). The hue and lightness of the two backgrounds are consistent with the product. Similar to Study 1, six colors (i.e., red, orange, yellow, green, blue, and purple) were selected (Hagtvedt and Adam Brasel, 2017; Wang et al., 2020). Thus, a total of 144 stimulus pairs (12 products × 12 presentation modes) were included in this experiment. All the S2 stimuli and their color specification related to Study 2 are provided in Supplementary Table 2. Throughout the experiment, each pair of stimuli was repeated twice and the size of each stimulus was 600 × 600 pixels.

### Experimental procedure

**FIGURE 5:**
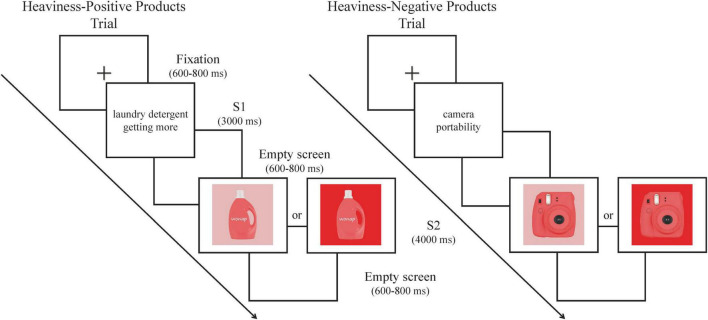
Experimental task: Participants were presented with four conditions of stimulus pairs. They were instructed to make a “buy” or “not buy” decision after the presentation of S2.

The experiment was conducted in a sound-attenuated room. Participants were asked to sit in a comfortable chair. The stimuli were presented on a 23-inch computer screen (resolution: 1,920 × 1,080 pixels) approximately placed 70 cm in front of the participant by using the E-Prime 3.0 software. The entire experiment consisted of 288 trials. To exclude the effects of hue, this experiment was divided into six blocks according to hue, with each block consisting of 48 trials. Each participant was informed that they could take a 2–3 min break between each block. The S1-S2 paradigm was applied in this experiment. As shown in Figure 5, each experiment began with a central fixed “+” for 600–800 ms, followed by a 3000 ms presentation of the product name and situational cues (S1). This was followed by an empty screen for 600–800 ms, and then a presentation of the product and its background (S2). After S2 was presented, participants were asked to make purchase decisions as quickly as possible within 4000 ms by using the keyboard (“f” for “buy”, “j” for “not buy”). After participants had pressed a button, the S2 stimuli disappeared. Then, an empty screen was randomly presented for 600–800 ms. Prior to the formal experiment, participants were instructed to read the experimental instructions and performed the eight practice trials.

### EEG recordings and analysis

According to the standard international 10–20 system, the EEG data were continuously recorded using an electrode cap with 32 Ag/AgCl electrodes with a sampling rate of 500 Hz and 0.05–100 Hz bandpass. Fz was chosen as the online reference, and electrode impedance of the scalp was maintained below 10 kΩ throughout the recording. The E-Prime 3.0 software was used to record participants’ behavioral data, including purchase rate (PR) and response times (RTs). We used the EEGLAB software to process offline EEG data. The raw data were re-referenced by the REST plugin and filtered by a low-pass filter at 30 Hz (Yao, 2001). The EEG was segmented from 200 ms before the start of S2 stimulus to 800 ms after this onset, with 200 ms before the S2 stimulus as the baseline. Artifacts embedded in the data (e.g., eye blink) were removed by applying independent component analysis (Semlitsch et al., 1986). In addition, any trials that exceeded ±75 uV were rejected. For each condition (heaviness-positive product-desaturated background, heaviness-positive product-saturated background, heaviness-negative product-desaturated background, and heaviness-negative product-saturated background), the EEG data were separately averaged for each participant, and then grand averaged.

As expected, the N2 and P3 components were induced by four conditions. Based on observation of the grand-averaged ERPs results and previous neuroscience studies, a time window of 200–270 ms was selected to analyze the mean N2 amplitude (Shang et al., 2017; Jing et al., 2019; Ma et al., 2021) and a time window of 310–460 ms was used to analyze the mean P3 amplitude (Fudali-Czyż et al., 2016; Shang et al., 2017; Song et al., 2022). For the N2 component, five electrodes distributed in the prefrontal-central region were selected for a 2 (product type: heaviness-positive product vs. heaviness-negative product) × 2 (presentation mode: saturated background vs. desaturated background) × 5 (electrode: F3, Fz, F4, FC1, and FC2) within-subjects repeated-measures ANOVA. Similarly, for the P3 component, five electrodes (CP1, CP2, P3, Pz, and P4) in the central-parietal area were selected for a 2 × 2 × 5 within-subjects repeated-measures ANOVA. The Greenhouse–Geisser correction (Greenhouse and Geisser, 1959) was used when the sphericity assumption was not applicable.

### Results

#### Behavioral data

**FIGURE 6:**
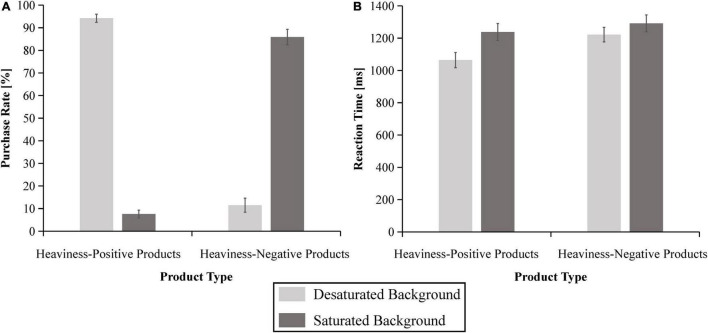
Behavioral results of the purchase rate **(A)** and the reaction times **(B)** for the four conditions. The error bars reflect the standard error of the mean.

A 2 (product type) × 2 (presentation mode) within-subjects repeated-measures ANOVA was carried out for PR and RTs. As for the PR, the results revealed significant main effects of product type [*F*(1,36) = 7.680, *p* < 0.01, $\eta_{P}^{2}$ = 0.176] and presentation mode [*F*(1,36) = 6.202, *p* < 0.05, $\eta_{P}^{2}$ = 0.147]. In addition, the interaction effects between them were also significant [*F*(1,36) = 324.647, *p* < 0.001, $\eta_{P}^{2}$ = 0.900], as illustrated in Figure 6A. A simple effect analysis suggested that the PR in the desaturated background condition (*M* = 0.942, SE = 0.018) was significantly higher than that in the saturated background condition (*M* = 0.076, SE = 0.017) for the heaviness-positive products [*F*(1,36) = 701.043, *p* < 0.001, $\eta_{P}^{2}$ = 0.951]. In contrast, the PR in the saturated background condition (*M* = 0.859, SE = 0.034) was significantly higher than that in the desaturated background condition (*M* = 0.115, SE = 0.031) for the heaviness-negative products [*F*(1,36) = 133.759, *p* < 0.001, $\eta_{P}^{2}$ = 0.788].

Regarding the RTs, the ANOVA results demonstrated significant main effects of product type [*F*(1,36) = 27.743, *p* < 0.001, $\eta_{P}^{2}$ = 0.435] and presentation mode [*F*(1,36) = 49.529, *p* < 0.001, $\eta_{P}^{2}$ = 0.579]. Furthermore, the interaction effects between them were significant [*F*(1,36) = 11.184, *p* < 0.01, $\eta_{P}^{2}$ = 0.237], as shown in Figure 6B. A simple effects analysis indicated that the RT in the desaturated background condition (*M* = 1064.150 ms, SE = 47.086) was significantly shorter than that in the saturated background condition (*M* = 1238.002 ms, SE = 52.494) for the heaviness-positive products [*F*(1,36) = 67.815, *p* < 0.001, $\eta_{P}^{2}$ = 0.653]. Notably, the RT in the saturated background condition (*M* = 1291.808 ms, SE = 52.194) was significantly longer than that in the desaturated background condition (*M* = 1221.983 ms, SE = 44.898) for the heaviness-negative products [*F*(1,36) = 7.648, *p* < 0.01, $\eta_{P}^{2}$ = 0.175].

#### Event-related potential data

**FIGURE 7:**
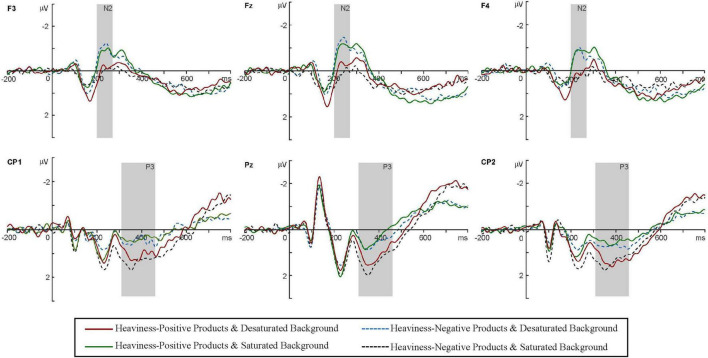
Grand-averaged ERPs of N2 and P3 for the four conditions at representative electrodes (F3, Fz, F4, CP1, Pz, and CP2).

**FIGURE 8:**
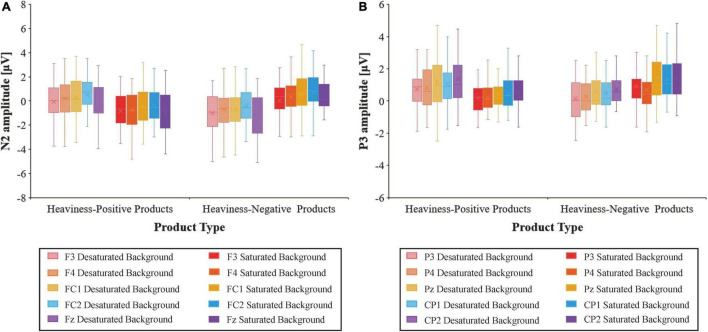
The box plots of the average amplitudes of the N2 **(A)** and P3 **(B)** components at the relevant electrodes for the four conditions.

**FIGURE 9:**
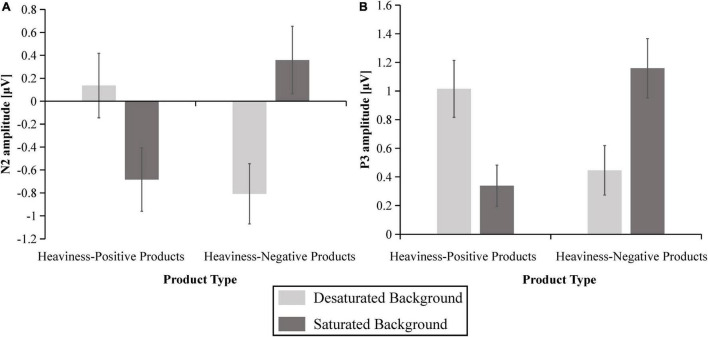
The mean amplitudes of N2 at F3/Fz/F4/FC1/FC2 **(A)**, and P3 at CP1/CP2/P3/Pz/P4 **(B)**. The error bars reflect the standard error of the mean.

Figure 7 indicates the grand-averaged ERP triggered by the four conditions. The box plots of the average amplitudes of the N2 and P3 components at the relevant electrodes for the four conditions are shown in Figure 8. For the N2, the results showed significant main effects of presentation mode [*F*(1,36) = 4.236, *p* < 0.05, $\eta_{P}^{2}$ = 0.105] and electrode [*F*(4,144) = 12.072, *p* < 0.001, $\eta_{P}^{2}$ = 0.251]. However, there were no significant main effects of product type [*F*(1,36) = 0.307, *p* > 0.05, $\eta_{P}^{2}$ = 0.008]. Additionally, the interaction between presentation mode and product type was significant [*F*(1,36) = 20.571, *p* < 0.001, $\eta_{P}^{2}$ = 0.364], as shown in Figure 9A. A simple effects analysis suggested that the N2 amplitudes in the saturated background condition (*M* = −0.684 μV, SE = 0.276) were more negative than those in the desaturated background condition (*M* = 0.136 μV, SE = 0.281) for the heaviness-positive products [*F*(1,36) = 11.761, *p* < 0.01, $\eta_{P}^{2}$ = 0.246]. Conversely, the N2 amplitudes in the desaturated background condition (*M* = −0.808 μV, SE = 0.263) were more negative than those in the saturated background condition (*M* = 0.359 μV, SE = 0.295) for the heaviness-negative products [*F*(1,36) = 25.735, *p* < 0.001, $\eta_{P}^{2}$ = 0.417]. Moreover, the interactions between presentation mode and electrode were significant [*F*(4,144) = 3.963, *p* < 0.05, $\eta_{P}^{2}$ = 0.099]. *Post hoc* comparisons indicated that the N2 at the FC1 and Fz electrodes was more negative in the desaturated background condition than in the saturated background condition. However, there were no significant differences at other electrodes (*p* > 0.05). As we expected, there were no interaction effects of product type × electrode [*F*(4,144) = 0.711, *p* > 0.05, $\eta_{P}^{2}$ = 0.019] or product type × presentation mode × electrode [*F*(4,144) = 0.348, *p* > 0.05, $\eta_{P}^{2}$ = 0.010].

With regard to the P3, the results indicated that the main effects of product type [*F*(1,36) = 1.744, *p* > 0.05, $\eta_{P}^{2}$ = 0.046] and presentation mode [*F*(1,36) = 0.026, *p* > 0.05, $\eta_{P}^{2}$ = 0.001] were not significant. However, there was a significant main effect of the electrode [*F*(4,144) = 5.125, *p* < 0.01, $\eta_{P}^{2}$ = 0.125]. Furthermore, the interaction between product type and presentation mode was significant [*F*(1,36) = 18.679, *p* < 0.001, $\eta_{P}^{2}$ = 0.342], as shown in Figure 9B. A simple effects analysis demonstrated that the P3 amplitudes in the desaturated background condition (*M* = 1.015 μV, SE = 0.199) were more positive than those in the saturated background condition (*M* = 0.338 μV, SE = 0.144) for the heaviness-positive products [*F*(1,36) = 12.978, *p* < 0.01, $\eta_{P}^{2}$ = 0.265]. On the contrary, the P3 amplitudes in the saturated background condition (*M* = 1.159 μV, SE = 0.207) were more positive than those in the desaturated background condition (*M* = 0.446 μV, SE = 0.173) for the heaviness-negative products [*F*(1,36) = 12.469, *p* < 0.01, $\eta_{P}^{2}$ = 0.257]. However, there were no interaction effects of product type × electrode [*F*(4,144) = 2.001, *p* > 0.05, $\eta_{P}^{2}$ = 0.053], presentation mode × electrode [*F*(4,144) = 1.443, *p* > 0.05, $\eta_{P}^{2}$ = 0.039] or product type × presentation mode × electrode [*F*(4,144) = 1.399, *p* > 0.05, $\eta_{P}^{2}$ = 0.037].

### Discussion

The main aim of Study 2 was to explore how product-background saturation combinations (desaturated background and saturated background) affect the neural basis of the consumer purchase decisions for the different product types (heaviness-positive products and heaviness-negative products). Both the behavioral and ERP results indicated that the heaviness-positive product-desaturated background and heaviness-negative product-saturated background conditions had a higher fit compared to the heaviness-positive product-saturated background and heaviness-negative product-desaturated background conditions, which facilitated consumer purchase decisions. Behavioral results suggested that the desaturated background enhanced the PR for the heaviness-positive products, while the opposite outcomes were realized for the heaviness-negative products. Furthermore, the shorter RTs were observed in the desaturated background condition for both the heaviness-positive and heaviness-negative products. The ERP results revealed that the desaturated background led to an attenuated N2 and a larger P3, in contrast to the saturated background for the heaviness-positive products, whereas the reverse held for the heaviness-negative products.

Behaviorally, we found a remarkable PR effect: the PR in the desaturated background condition was higher than that in the saturated background condition for the heaviness-positive products. However, the reverse held for the heaviness-negative products. An increased heaviness perception has been shown to enhance consumer preference for the heaviness-positive products. Sharma and Romero (2020) found that a product with a shadow is considered to be heavier than the identical product without a shadow. Thus, when faced with the heaviness-positive products (e.g., vitamin supplements), consumers show a higher preference for the product with a shadow. In contrast, the increased perception of product heaviness produces a negative effect on the heaviness-negative products. For instance, in the case of mobile phones (heaviness-negative products), higher visual densities increase the perception of product heaviness and reduce consumer product evaluation (Choe et al., 2021). In this study, the desaturated background increased the perceived heaviness of the product compared to the saturated background. Therefore, the desaturated background enhances consumers’ purchase intentions compared to the saturated background for the heaviness-positive products. Instead, the opposite results were observed for the heaviness-negative products.

With regard to the RTs, less decision time was spent in the desaturated background condition than in the desaturated background condition for the heaviness-positive products. The RTs have been associated with task difficulty and cognitive load. More specifically, a shorter reaction time typically represents a lower cognitive load and a smaller task difficulty (Sweller, 1988; Wang et al., 2016; Jin et al., 2017). Ma et al. (2018), for example, demonstrated that zero price conditions were more consistent with participants’ expectations than normal price conditions, resulting in decreased decision difficulty and reduced decision time. In the present study, the desaturated background was more consistent with participants’ expectations than the saturated background for the heaviness-positive products, which led to an easier purchase decision. As a result, the desaturated background contributed to a shorter decision time than the saturated background. However, the RT in the desaturated background condition was still shorter than that in the saturated background condition for the heaviness-negative products. Previous research has illustrated that contrast can be improved in advertising by using white space to separate key advertising elements from the background (Pracejus et al., 2006; Olsen et al., 2012; Kwan et al., 2017). Extending this conclusion to our study, the contrast in the desaturated background condition was greater than that in the saturated background condition. Further, previous research has indicated that higher contrast will help consumers to identify key messages in advertisements, which will reduce their cognitive load (Pieters et al., 2010; Kim and Lakshmanan, 2015). According to this logic, we argue that greater contrast in the desaturated background condition can reduce the consumers’ cognitive load in the decision process relative to the saturated background condition. Therefore, the RTs in the desaturated background condition were shorter than in the saturated background condition for the heaviness-negative products.

Regarding the ERPs component, the differences in N2 amplitudes were observed for four conditions within a time window of 200–270 ms. N2 has been positively correlated with perception conflict (Folstein and Van Petten, 2007; Shang et al., 2017). Jing et al. (2019) suggested that participants use low price or small purchase volumes as a criterion when purchasing hedonic products. Consequently, in the condition of deep discounts, the quantity promotion did not match consumers’ expectations when compared to the price promotion, eliciting greater perceived conflict and a more negative N2 amplitude. Moreover, the perceived heaviness of a product is often used as a standard when consumers purchase products with different heaviness attributes. The product is considered to be heavier when the product image is placed at a heavy location on the package, which matches consumers’ expectations of the heaviness-positive products and increases consumer preference (Deng and Kahn, 2009). In the current study, different presentation modes resulted in differences in the perceived heaviness and influenced consumer preferences for different product types. More specifically, the presentation mode in the desaturated background condition made the product appear heavier and fit participants’ expectations of the heaviness-positive products, while the presentation mode in the saturated background condition made the product appear lighter and fit participants’ expectations of the heaviness-negative products. Thus, the saturated background elicited greater perception conflict and a more negative N2 amplitude for the heaviness-positive products compared to the desaturated background, while the reverse held for the heaviness-negative products.

Moreover, the differences in P3 amplitudes were found for four conditions within a time window of 310–460 ms. Previous research has revealed that the more similar the target is to a previous stimulus or working memory, the greater the P3 amplitude (Azizian et al., 2006; Ma et al., 2008). Research on brand extensions suggested that participants do not have stereotypical impressions of brand logos in long-term memory compared to brand names. Therefore, participants showed higher perceived similarity and greater P3 amplitudes when facing brand logo extension strategies for different product categories (Shang et al., 2017). The two stimuli (S1 and S2) were sequentially presented in the current study. For the heaviness-positive products, participants formed the expectation that the heavier, the better based on the information presented in S1. When the information about heaviness-positive product-desaturated background presented in S2 was transmitted to the brain, participants perceived a greater similarity, as reflected by a more positive P3 amplitude. Contrastingly, they perceived a lower similarity when faced with the heaviness-positive product-saturated background information, as evidenced by a smaller P3 amplitude. The opposite results were observed for the heaviness-negative products. In addition, P3 was associated with decision difficulty and decision confidence. The decreased P3 component was found when consumers faced greater decision difficulty or lack of confidence (Qin et al., 2009; Wang et al., 2022). Gong et al. (2018) found that compared to discount promotions, gift-giving promotions can result in increased decision difficulties and reduced decision confidence, and then a less positive P3 can be observed. In the current study, the desaturated background elicited a larger P3 amplitude than the saturated background for the heaviness-positive products, while the saturated background elicited a larger P3 amplitude than the desaturated background for the heaviness-negative products. These findings indicate that consumers perceive less decision difficulties and become more confident when the visuals presented in the presentation mode are consistent with their expectations (i.e., heaviness-positive product-desaturated background and heaviness-negative product-saturated background).

## General discussion

### Theoretical contributions

Based on the color simultaneous contrast theory, we investigated the influence of product-background saturation combinations on consumer decisions and uncovered the underlying neural mechanisms. Theoretical contributions of this study contain the following main aspects. Firstly, despite the increasing attention to color research in marketing, research concerning the effects of color contrast on consumer behaviors are still limited. To fill this gap, we explored the impact of simultaneous saturation contrast on consumers by using ERPs. Secondly, previous research on color marketing has only focused on the influence of a single carrier’s color on consumer decisions (Bagchi and Cheema, 2013; Hagtvedt and Brasel, 2016; Wang et al., 2020). In daily life, however, the carriers of color (product and background) are often presented simultaneously and develop joint visual effects to impact consumer behaviors (Huang et al., 2020; Jeon et al., 2020). The current study applied the simultaneous saturation contrast to product and background color combinations and explored the effects of the interactions between product-background color combinations and product types on consumer behaviors. Finally, the current study investigated the influence of simultaneous saturation contrast on heaviness perceptions, which expands the visual cues that affect consumer product heaviness perceptions (e.g., completeness, product image location, and shadow) (Deng and Kahn, 2009; Sevilla and Kahn, 2014; Sharma and Romero, 2020). Moreover, we examined how the increased perceived heaviness of the product affects the downstream consequences.

### Practical implications

Our work provides marketers with several enforceable insights. Based on our findings, there is no one product presentation mode that matches all product types. Therefore, when designing product presentation modes, marketers should consider the characteristics of different product types. Specifically, for the heaviness-positive products, marketers should design a desaturated background to enhance the perceived heaviness of the product; in contrast, for the heaviness-negative products, marketers should design a saturated background to reduce the perceived heaviness of the product. Additionally, our findings suggest that product presentation mode can better communicate the heaviness of a product to consumers in e-commerce marketing. Importantly, unlike previous studies (Hagtvedt and Adam Brasel, 2017; Hagtvedt, 2020; Wang et al., 2020), our study alters the visual heaviness perception of the product by manipulating the background saturation without altering the original product or package. Therefore, our findings provide references for online retailers and marketers.

### Limitations and future research

However, we must acknowledge that there are some limitations in this study. Firstly, the various color dimensions do not exist in isolation. This study only explored the effects of saturation combinations on heaviness perception and their downstream outcomes. However, it has been reported that product lightness affects product heaviness perception and participants’ preferences (Hagtvedt, 2020). Therefore, future research should investigate the effects of the combination between various dimensions on the heaviness perception and downstream outcomes. Secondly, previous studies have shown that factors such as position (Deng and Kahn, 2009), speed (Jia et al., 2020), or sound (Takashima, 2018) can produce an impact on the perceived heaviness. We believe that future research can investigate saturation combinations in conjunction with other visual factors, such as location or speed. Even the effects of sensory stimuli combinations (e.g., sight and sound) on consumer behaviors can be examined. Thirdly, our study merely demonstrates the impact of product-background color combinations on consumer behaviors. We recommend that future research should explore the effects of other color carrier combinations (e.g., product and surroundings) to prove the generalizability of our study. Fourthly, in Study 2, data from 37 valid participants were analyzed. For the N2, 31 participants showed a pattern of results that was consistent with the grand mean. For the P3, 32 participants showed a pattern of results that was consistent with the grand mean. It is possible that a minority of participants concentrated more on the desaturated background condition to overcome the reduced visibility of the product and showed the opposite pattern. Since ERP experiments have rigorous rules for the stimuli. The products and their backgrounds (presentation modes) were simplified in Study 2 compared to real-life decision making. Future research should adopt more advanced techniques to process experimental stimuli to ensure the same visibility of all material. Finally, our study only focused on the factor of product-background color combinations without considering other marketing mix. One of the key factors is the brand concepts communicated by companies, which have been proved to be of great importance to consumer decisions (He et al., 2018). For example, in the context of anti-globalization, some brands try to convey a sense of “localness” with the purpose of attracting the patriots (Steenkamp, 2019). On the contrary, some brands strive to associate their concepts with internationalism and cosmopolitanism (Huang and He, 2019), representing widely accepted world views (e.g., world-wide empathy, compassion, and cooperation). However, no research has explored how consumers respond to the two different brand concepts using neuroscience methods. We believe exploring such issues from the perspective of neuroscience would be more theoretically significant in the context of anti-globalization.

## Conclusion

Taken together, this study explored how product-background color combinations affect consumer behaviors for products with different heaviness attributes. Study 1 investigated the effects of simultaneous contrast of saturation on heaviness perception. The results showed that the patch of color surrounded by the desaturated background was perceived as visually heavier compared to the saturated background. Based on ERPs, Study 2 investigated the influence of the interactions between product-background color combinations and product types on consumer purchase behaviors and its neural mechanisms. Behavioral results indicated that the desaturated background led to a higher PR compared to the saturated background for the heaviness-positive products. The results were reversed for the heaviness-negative products. Furthermore, a shorter RT was observed in the desaturated background condition rather than the saturated background condition for both the heaviness-positive and heaviness-negative products. The ERP results indicated that the desaturated background elicited an attenuated N2 and an increased P3 compared to the saturated background for the heaviness-positive products, whereas the opposite outcome was realized for the heaviness-negative products. We argue that N2 represents a cognitive conflict between presentation mode and product type, and P3 reflects decision difficulty and decision confidence. In summary, our study demonstrates that if the product presentation mode fits the product type, such a matching effect facilitates consumers’ purchase decisions. Our findings provide beneficial insights for marketing research and practice.

## Data availability statement

The raw data supporting the conclusions of this article will be made available by the authors, without undue reservation.

## Ethics statement

The studies involving human participants were reviewed and approved by the School of Economics and Management, Wuyi University. The patients/participants provided their written informed consent to participate in this study.

## Author contributions

MP, YT, and HH conceived and designed the two experiments. YT and LJ performed the behavioral experiment. MP, YT, ZX, and HH performed the ERP experiment. YT and ZX analyzed the data. All authors wrote and refined the manuscript, contributed to the article, and approved the submitted version.
