# Supplementary material for: How does the use of simultaneous contrast illusion on product-background color combination nudge consumer behavior? A behavioral and event-related potential study
Source: Front Neurosci. 2022 Jul 27;16:942901. doi: 10.3389/fnins.2022.942901 (PMC9363632; doi:10.3389/fnins.2022.942901)
Supplement: Supplementary file 1 [file Data_Sheet_1.PDF]

Supplementary Material

1 Supplementary Figures and Tables

1.1 Supplementary Tables

For Studies 1 and 2, the values of the three dimensions of color were adapted from Hagtvedt and Brasel (2017) and Wang et al. (2020). In addition, we used Adobe Photoshop to manufacture all the stimuli following the HSV model. All the stimuli and their color specification are presented in Table 1 and Table 2, respectively.

Supplementary Table 1. Color specifications for all stimuli in Study 1

	Hue	Saturation	H	S	V	R	G	B
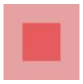	Red	Color patch	0	60	90	229.5	91.8	91.8
		Desaturated	0	30	90	229.5	160.65	160.65
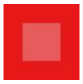		Saturated	0	90	90	229.5	22.95	22.95
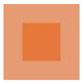	Orange	Color patch	22	75	90	229.5	120.49	57.38
		Desaturated	22	50	90	229.5	156.82	114.75
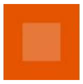		Saturated	22	100	90	229.5	84.15	0
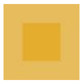	Yellow	Color patch	42	80	90	229.5	174.42	45.9
		Desaturated	42	60	90	229.5	188.19	91.8
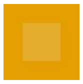		Saturated	42	100	90	229.5	160.65	0
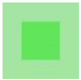	Green	Color patch	117	60	90	98.69	229.5	91.8
		Desaturated	117	30	90	164.09	229.5	160.65
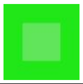		Saturated	117	90	90	33.28	229.5	22.95

(continues)

Supplementary Table 1. (continued)

	Hue	Saturation	H	S	V	R	G	B
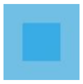		Color patch	200	75	90	57.38	172.13	229.5
	Blue	Desaturated	200	50	90	114.75	191.25	229.5
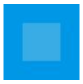		Saturated	200	100	90	0	153	229.5
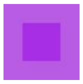		Color patch	280	80	90	168.3	45.9	229.5
	Purple	Desaturated	280	60	90	183.6	91.8	229.5
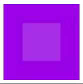		Saturated	280	100	90	153	0	229.5

Supplementary Table 2. Color specifications for all S2 stimuli in Study 2

	**Hue**	**Saturation**	**H**	**S**	**V**	**R**	**G**	**B**
Laundry detergent								
		Product	0	60	90	229.5	91.8	91.8
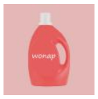 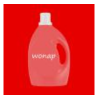	Red	Desaturated	0	20	90	229.5	183.6	183.6
		Saturated	0	100	90	229.5	0	0
		Product	22	60	90	229.5	142.29	91.8
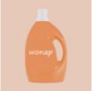 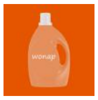	Orange	Desaturated	22	20	90	229.5	200.43	183.6
		Saturated	22	100	90	229.5	84.15	0
		Product	42	60	90	229.5	188.19	91.8
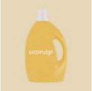 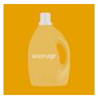	Yellow	Desaturated	42	20	90	229.5	215.73	183.6
		Saturated	42	100	90	229.5	160.65	0
		Product	142	60	90	91.8	229.5	142.29
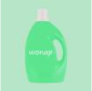 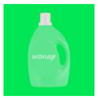	Green	Desaturated	142	20	90	183.6	229.5	200.43
		Saturated	142	100	90	0	229.5	84.15
		Product	200	60	90	91.8	183.6	229.5
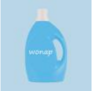 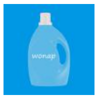	Blue	Desaturated	200	20	90	183.6	214.2	229.5
		Saturated	200	100	90	0	153	229.5
		Product	280	60	90	183.6	91.8	229.5
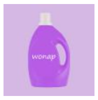 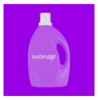	Purple	Desaturated	280	20	90	214.2	183.6	229.5
		Saturated	280	100	90	153	0	229.5

(continues)

Supplementary Table 2. (continued)

	Hue	Saturation	H	S	V	R	G	B
Shampoo								
		Product	0	60	90	229.5	91.8	91.8
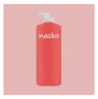 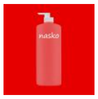	Red	Desaturated	0	20	90	229.5	183.6	183.6
		Saturated	0	100	90	229.5	0	0
		Product	22	60	90	229.5	142.29	91.8
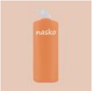 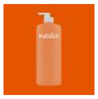	Orange	Desaturated	22	20	90	229.5	200.43	183.6
		Saturated	22	100	90	229.5	84.15	0
		Product	42	60	90	229.5	188.19	91.8
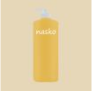 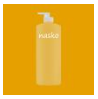	Yellow	Desaturated	42	20	90	229.5	215.73	183.6
		Saturated	42	100	90	229.5	160.65	0
		Product	142	60	90	91.8	229.5	142.29
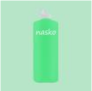 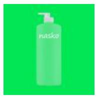	Green	Desaturated	142	20	90	183.6	229.5	200.43
		Saturated	142	100	90	0	229.5	84.15
		Product	200	60	90	91.8	183.6	229.5
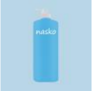 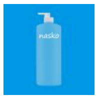	Blue	Desaturated	200	20	90	183.6	214.2	229.5
		Saturated	200	100	90	0	153	229.5
		Product	280	60	90	183.6	91.8	229.5
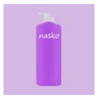 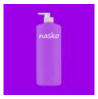	Purple	Desaturated	280	20	90	214.2	183.6	229.5
		Saturated	280	100	90	153	0	229.5

(continues)

Supplementary Table 2. (continued)

	Hue	Saturation	H	S	V	R	G	B
Beverage								
		Product	0	60	90	229.5	91.8	91.8
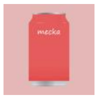 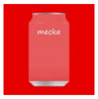	Red	Desaturated	0	20	90	229.5	183.6	183.6
		Saturated	0	100	90	229.5	0	0
		Product	22	60	90	229.5	142.29	91.8
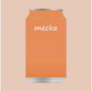 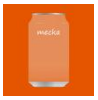	Orange	Desaturated	22	20	90	229.5	200.43	183.6
		Saturated	22	100	90	229.5	84.15	0
		Product	42	60	90	229.5	188.19	91.8
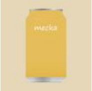 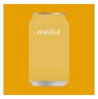	Yellow	Desaturated	42	20	90	229.5	215.73	183.6
		Saturated	42	100	90	229.5	160.65	0
		Product	142	60	90	91.8	229.5	142.29
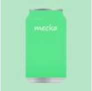 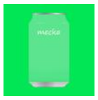	Green	Desaturated	142	20	90	183.6	229.5	200.43
		Saturated	142	100	90	0	229.5	84.15
		Product	200	60	90	91.8	183.6	229.5
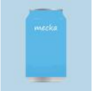 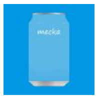	Blue	Desaturated	200	20	90	183.6	214.2	229.5
		Saturated	200	100	90	0	153	229.5
		Product	280	60	90	183.6	91.8	229.5
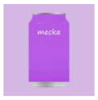 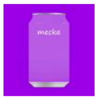	Purple	Desaturated	280	20	90	214.2	183.6	229.5
		Saturated	280	100	90	153	0	229.5

(continues)

Supplementary Table 2. (continued)

	Hue	Saturation	H	S	V	R	G	B
Potato chips								
		Product	0	60	90	229.5	91.8	91.8
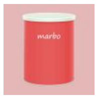 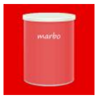	Red	Desaturated	0	20	90	229.5	183.6	183.6
		Saturated	0	100	90	229.5	0	0
		Product	22	60	90	229.5	142.29	91.8
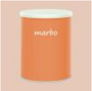 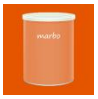	Orange	Desaturated	22	20	90	229.5	200.43	183.6
		Saturated	22	100	90	229.5	84.15	0
		Product	42	60	90	229.5	188.19	91.8
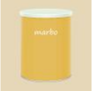 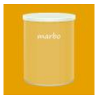	Yellow	Desaturated	42	20	90	229.5	215.73	183.6
		Saturated	42	100	90	229.5	160.65	0
		Product	142	60	90	91.8	229.5	142.29
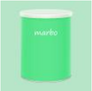 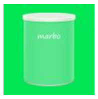	Green	Desaturated	142	20	90	183.6	229.5	200.43
		Saturated	142	100	90	0	229.5	84.15
		Product	200	60	90	91.8	183.6	229.5
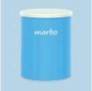 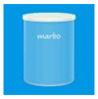	Blue	Desaturated	200	20	90	183.6	214.2	229.5
		Saturated	200	100	90	0	153	229.5
		Product	280	60	90	183.6	91.8	229.5
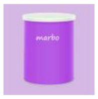 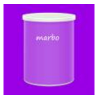	Purple	Desaturated	280	20	90	214.2	183.6	229.5
		Saturated	280	100	90	153	0	229.5

(continues)

Supplementary Table 2. (continued)

	Hue	Saturation	H	S	V	R	G	B
Suitcase								
		Product	0	60	90	229.5	91.8	91.8
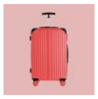 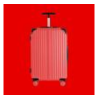	Red	Desaturated	0	20	90	229.5	183.6	183.6
		Saturated	0	100	90	229.5	0	0
		Product	22	60	90	229.5	142.29	91.8
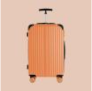 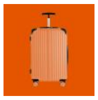	Orange	Desaturated	22	20	90	229.5	200.43	183.6
		Saturated	22	100	90	229.5	84.15	0
		Product	42	60	90	229.5	188.19	91.8
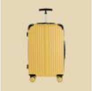 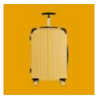	Yellow	Desaturated	42	20	90	229.5	215.73	183.6
		Saturated	42	100	90	229.5	160.65	0
		Product	142	60	90	91.8	229.5	142.29
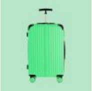 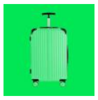	Green	Desaturated	142	20	90	183.6	229.5	200.43
		Saturated	142	100	90	0	229.5	84.15
		Product	200	60	90	91.8	183.6	229.5
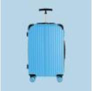 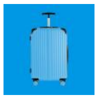	Blue	Desaturated	200	20	90	183.6	214.2	229.5
		Saturated	200	100	90	0	153	229.5
		Product	280	60	90	183.6	91.8	229.5
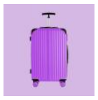 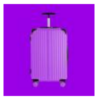	Purple	Desaturated	280	20	90	214.2	183.6	229.5
		Saturated	280	100	90	153	0	229.5

(continues)

Supplementary Table 2. (continued)

	Hue	Saturation	H	S	V	R	G	B
Washing machine								
		Product	0	60	90	229.5	91.8	91.8
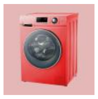 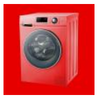	Red	Desaturated	0	20	90	229.5	183.6	183.6
		Saturated	0	100	90	229.5	0	0
		Product	22	60	90	229.5	142.29	91.8
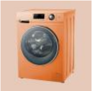 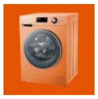	Orange	Desaturated	22	20	90	229.5	200.43	183.6
		Saturated	22	100	90	229.5	84.15	0
		Product	42	60	90	229.5	188.19	91.8
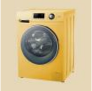 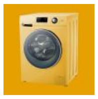	Yellow	Desaturated	42	20	90	229.5	215.73	183.6
		Saturated	42	100	90	229.5	160.65	0
		Product	142	60	90	91.8	229.5	142.29
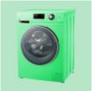 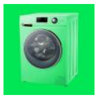	Green	Desaturated	142	20	90	183.6	229.5	200.43
		Saturated	142	100	90	0	229.5	84.15
		Product	200	60	90	91.8	183.6	229.5
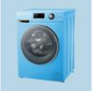 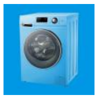	Blue	Desaturated	200	20	90	183.6	214.2	229.5
		Saturated	200	100	90	0	153	229.5
		Product	280	60	90	183.6	91.8	229.5
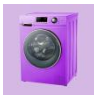 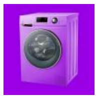	Purple	Desaturated	280	20	90	214.2	183.6	229.5
		Saturated	280	100	90	153	0	229.5

(continues)

Supplementary Table 2. (continued)

	Hue	Saturation	H	S	V	R	G	B
Kettle								
		Product	0	60	90	229.5	91.8	91.8
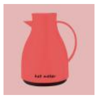 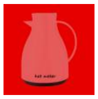	Red	Desaturated	0	20	90	229.5	183.6	183.6
		Saturated	0	100	90	229.5	0	0
		Product	22	60	90	229.5	142.29	91.8
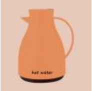 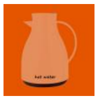	Orange	Desaturated	22	20	90	229.5	200.43	183.6
		Saturated	22	100	90	229.5	84.15	0
		Product	42	60	90	229.5	188.19	91.8
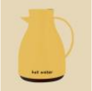 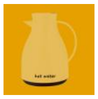	Yellow	Desaturated	42	20	90	229.5	215.73	183.6
		Saturated	42	100	90	229.5	160.65	0
		Product	142	60	90	91.8	229.5	142.29
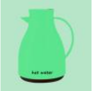 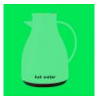	Green	Desaturated	142	20	90	183.6	229.5	200.43
		Saturated	142	100	90	0	229.5	84.15
		Product	200	60	90	91.8	183.6	229.5
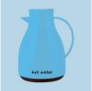 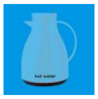	Blue	Desaturated	200	20	90	183.6	214.2	229.5
		Saturated	200	100	90	0	153	229.5
		Product	280	60	90	183.6	91.8	229.5
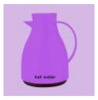 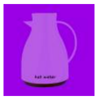	Purple	Desaturated	280	20	90	214.2	183.6	229.5
		Saturated	280	100	90	153	0	229.5

Supplementary Table 2. (continued)

		Hue	Saturation	H	S	V	R	G	B
Lamps									
			Product	0	60	90	229.5	91.8	91.8
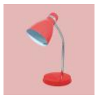	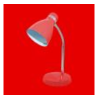	Red	Desaturated	0	20	90	229.5	183.6	183.6
			Saturated	0	100	90	229.5	0	0
			Product	22	60	90	229.5	142.29	91.8
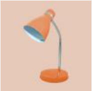	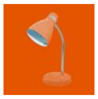	Orange	Desaturated	22	20	90	229.5	200.43	183.6
			Saturated	22	100	90	229.5	84.15	0
			Product	42	60	90	229.5	188.19	91.8
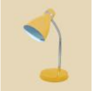	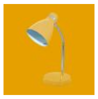	Yellow	Desaturated	42	20	90	229.5	215.73	183.6
			Saturated	42	100	90	229.5	160.65	0
			Product	142	60	90	91.8	229.5	142.29
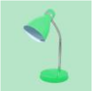	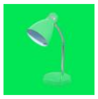	Green	Desaturated	142	20	90	183.6	229.5	200.43
			Saturated	142	100	90	0	229.5	84.15
			Product	200	60	90	91.8	183.6	229.5
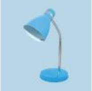	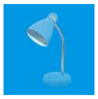	Blue	Desaturated	200	20	90	183.6	214.2	229.5
			Saturated	200	100	90	0	153	229.5
			Product	280	60	90	183.6	91.8	229.5
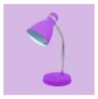	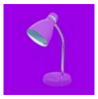	Purple	Desaturated	280	20	90	214.2	183.6	229.5
			Saturated	280	100	90	153	0	229.5

(continues)

Supplementary Table 2. (continued)

	Hue	Saturation	H	S	V	R	G	B
Laptop								
		Product	0	60	90	229.5	91.8	91.8
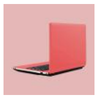 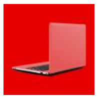	Red	Desaturated	0	20	90	229.5	183.6	183.6
		Saturated	0	100	90	229.5	0	0
		Product	22	60	90	229.5	142.29	91.8
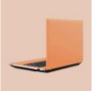 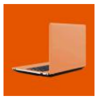	Orange	Desaturated	22	20	90	229.5	200.43	183.6
		Saturated	22	100	90	229.5	84.15	0
		Product	42	60	90	229.5	188.19	91.8
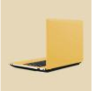 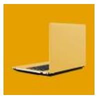	Yellow	Desaturated	42	20	90	229.5	215.73	183.6
		Saturated	42	100	90	229.5	160.65	0
		Product	142	60	90	91.8	229.5	142.29
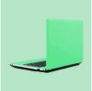 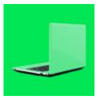	Green	Desaturated	142	20	90	183.6	229.5	200.43
		Saturated	142	100	90	0	229.5	84.15
		Product	200	60	90	91.8	183.6	229.5
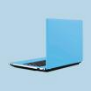 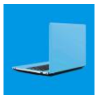	Blue	Desaturated	200	20	90	183.6	214.2	229.5
		Saturated	200	100	90	0	153	229.5
		Product	280	60	90	183.6	91.8	229.5
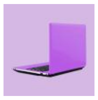 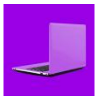	Purple	Desaturated	280	20	90	214.2	183.6	229.5
		Saturated	280	100	90	153	0	229.5

(continues)

Supplementary Table 2. (continued)

	Hue	Saturation	H	S	V	R	G	B
Speaker								
		Product	0	60	90	229.5	91.8	91.8
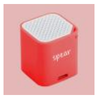 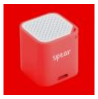	Red	Desaturated	0	20	90	229.5	183.6	183.6
		Saturated	0	100	90	229.5	0	0
		Product	22	60	90	229.5	142.29	91.8
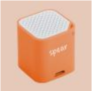 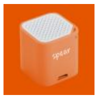	Orange	Desaturated	22	20	90	229.5	200.43	183.6
		Saturated	22	100	90	229.5	84.15	0
		Product	42	60	90	229.5	188.19	91.8
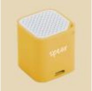 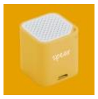	Yellow	Desaturated	42	20	90	229.5	215.73	183.6
		Saturated	42	100	90	229.5	160.65	0
		Product	142	60	90	91.8	229.5	142.29
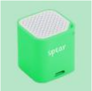 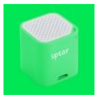	Green	Desaturated	142	20	90	183.6	229.5	200.43
		Saturated	142	100	90	0	229.5	84.15
		Product	200	60	90	91.8	183.6	229.5
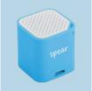 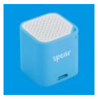	Blue	Desaturated	200	20	90	183.6	214.2	229.5
		Saturated	200	100	90	0	153	229.5
		Product	280	60	90	183.6	91.8	229.5
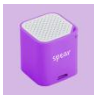 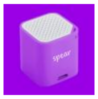	Purple	Desaturated	280	20	90	214.2	183.6	229.5
		Saturated	280	100	90	153	0	229.5

(continues)

Supplementary Table 2. (continued)

	Hue	Saturation	H	S	V	R	G	B
Camera								
		Product	0	60	90	229.5	91.8	91.8
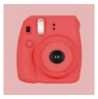 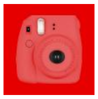	Red	Desaturated	0	20	90	229.5	183.6	183.6
		Saturated	0	100	90	229.5	0	0
		Product	22	60	90	229.5	142.29	91.8
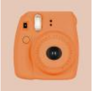 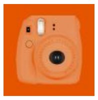	Orange	Desaturated	22	20	90	229.5	200.43	183.6
		Saturated	22	100	90	229.5	84.15	0
		Product	42	60	90	229.5	188.19	91.8
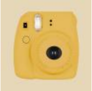 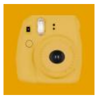	Yellow	Desaturated	42	20	90	229.5	215.73	183.6
		Saturated	42	100	90	229.5	160.65	0
		Product	142	60	90	91.8	229.5	142.29
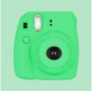 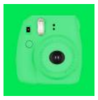	Green	Desaturated	142	20	90	183.6	229.5	200.43
		Saturated	142	100	90	0	229.5	84.15
		Product	200	60	90	91.8	183.6	229.5
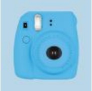 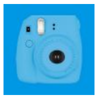	Blue	Desaturated	200	20	90	183.6	214.2	229.5
		Saturated	200	100	90	0	153	229.5
		Product	280	60	90	183.6	91.8	229.5
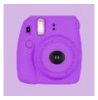 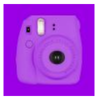	Purple	Desaturated	280	20	90	214.2	183.6	229.5
		Saturated	280	100	90	153	0	229.5

(continues)

Supplementary Table 2. (continued)

	Hue	Saturation	H	S	V	R	G	B
Rucksack								
		Product	0	60	90	229.5	91.8	91.8
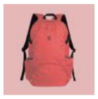 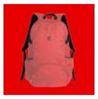	Red	Desaturated	0	20	90	229.5	183.6	183.6
		Saturated	0	100	90	229.5	0	0
		Product	22	60	90	229.5	142.29	91.8
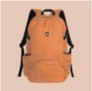 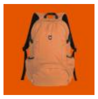	Orange	Desaturated	22	20	90	229.5	200.43	183.6
		Saturated	22	100	90	229.5	84.15	0
		Product	42	60	90	229.5	188.19	91.8
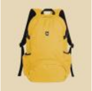 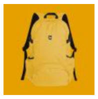	Yellow	Desaturated	42	20	90	229.5	215.73	183.6
		Saturated	42	100	90	229.5	160.65	0
		Product	142	60	90	91.8	229.5	142.29
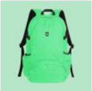 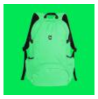	Green	Desaturated	142	20	90	183.6	229.5	200.43
		Saturated	142	100	90	0	229.5	84.15
		Product	200	60	90	91.8	183.6	229.5
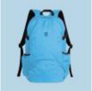 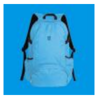	Blue	Desaturated	200	20	90	183.6	214.2	229.5
		Saturated	200	100	90	0	153	229.5
		Product	280	60	90	183.6	91.8	229.5
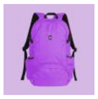 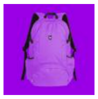	Purple	Desaturated	280	20	90	214.2	183.6	229.5
		Saturated	280	100	90	153	0	229.5
